# The elephant in the room: Family engagement in mental health and substance use research

**DOI:** 10.1111/hex.13827

**Published:** 2023-08-04

**Authors:** Lisa D. Hawke, Connie Putterman, Nathan Dawthorne, Shannon Pascoe, Shaylene Pind

**Affiliations:** ^1^ Centre for Addiction and Mental Health Toronto Ontario Canada; ^2^ University of Toronto Toronto Ontario Canada

## 1 INTRODUCTION

There is a growing emphasis in academic research on engaging people with lived experience (PWLE) of mental health and/or substance use challenges in research projects.^1^ People and communities with lived experience can be included in all aspects of research processes, which is increasingly encouraged by funding bodies and institutions. While engagement has grown rapidly in recent years,^2^ the movement is built upon decades of progressive experience in decentring academic work across disciplines through key informant collaboration. Lived experience engagement in research, also known as ‘patient engagement’ or ‘patient and public involvement’, provides many benefits to the research process, as PWLE are subject‐matter experts and key stakeholders. PWLE engagement occurs across the health disciplines, in a wide variety of research designs, including an extensive body of mental health and substance use research.^2^ Engaging PWLE promotes the inclusion of perspectives that matter, and stimulates the healing of the injustices of past and current imbalances and inequities in health care and research settings. To ensure that engagement is meaningful and not tokenistic, and thus remains ethically conscious, it is important to reflect on the conceptualization of engagement: what is engagement, who is engaged, and why?^3^

Amongst definitions of PWLE, family members or caregivers of people with mental health or substance use challenges are often included.^4^ Families can offer a holistic view of a person's life, observing long‐term trends in behaviour, as well as baselines, relationships and a historical synopsis of attempted treatment and self‐management strategies and interventions, helping to bridge the gaps in self‐reported measures of their loved ones. Family members can provide insights about the experience of living with someone with mental health or substance use challenges, caring for them, advocating for them, amplifying their voices and supporting them in their service‐seeking journeys, and about their own roles in family‐centred care. Supportive families can contribute to recovery and may play an important role in system navigation. Engaging family members in research gives them the opportunity to have their voices heard and to help make changes for other families. In areas of health in which the caregiving role is substantial or PWLE inputs may sometimes be limited, such as infant or early childhood mental health, or dementia, family members are often the primary engagement target and informants. However, across mental health and substance use more broadly, family members are often secondary to the engagement of people with direct, personal lived experience, even though they may fill a primary caregiving role.

While the general principles of engagement are similar across areas of health,^4^ there are considerations specific to mental health and substance use, and particular considerations when engaging families. Engagement in research can have many positive impacts.^2^ However it can also have negative impacts when conducted tokenistically or otherwise inappropriately, through erasure, marginalization, and stigmatization. Family members, who may feel marginalized, excluded, and even traumatized as they navigate the mental healthcare system with their loved one, can be further marginalized, excluded, and traumatized if they are engaged tokenistically,^5^ doing more harm than good as they relive their traumas. Other factors important to consider in family engagement include complex family dynamics and the impact of stigma and marginalization, alongside the many barriers to effective lived experience engagement.^2^ For family members, as for PWLE, it is important to conduct engagement in ways that are genuine, accessible, inclusive, destigmatizing, empathetic and trauma‐informed.^6^ However, there is a dearth of literature specific to family engagement in mental health and substance use research.

Given the complexities of family engagement in mental health and substance use research and the potential for tokenistic and unhelpful engagement, researchers are encouraged to reflect upon their motivations to engage families. Rather than engaging without reflection, because engagement represents a growing movement, they are advised to carefully consider the characteristics of their study and their target population to determine whether family engagement is appropriate for a given study. To aid in this decision, we propose a number of reflection points, described below and represented in Table 1. Key considerations include: (1) the relevance of the research question and study design to family members, (2) the representativeness of families and (3) whether family engagement is welcomed by all stakeholders. These reflections can guide a researcher's decision about when and whether to engage families, within the context of the study at hand. The decision points are not fast rules, but general reflection points and suggestions. A single response in the ‘*Consider not engaging families*’ category (Table 1) does not definitively preclude family engagement, but signals the need to reflect carefully on whether, why, and how family engagement might be appropriate, or inappropriate, for the study at hand. Multiple such responses suggest that family engagement may not be appropriate for the study. However, if formal family engagement is opted against in a given study, researchers might consider other creative ways to access family perspectives as part of their work.

**Table 1:** Areas of reflection when deciding whether to engage families in mental health and substance use research.

Aspect of the research and engagement context to consider			Consider engaging families	Consider not engaging families
Relevance of the research question and study design to families	The research is about family experiences or perspectives, or family‐centred care, or care that was codesigned with families.	Yes	✓	
		No		✓
	Families are participants in the study.	Yes	✓	
		No		✓
	PWLE are able to represent themselves fully in response to the research question.	Yes		✓
		No	✓	
Representativeness of families	The supportive families available for engagement are representative of the target population.	Yes	✓	
		No		✓
	The target population is highly affected by family violence, family trauma, or high levels of family conflict.	Yes		✓
		No	✓	
Family is welcomed by all stakeholders	Researchers have reflected on any biases they may have against families and recognize a genuine benefit of having family members at the table.	Yes	✓	
		No		✓
	The research team has specific questions for family members and is willing to directly incorporate their feedback.	Yes	✓	
		No		✓
	The PWLE engaged support a family engagement component.	Yes	✓	
		No		✓

Abbreviation: PWLE, people with lived experience of mental health or substance use challenges.

## 2 RELEVANCE OF THE RESEARCH QUESTION AND STUDY DESIGN TO FAMILY MEMBERS

Consistent with the purposes of authentic engagement, family members can be productively engaged when the research is directly relevant to them, in a manner that creates a shared purpose of work on the topic of the research. While some research in the mental health and substance use sphere directly addresses the experiences and perspectives of families or caregivers, such as family‐centred treatment research and family‐centred research designs, much of it does not. If a research question directly addresses family or caregiver experiences or roles, the research is testing an intervention for or with family members, it includes family members as study participants or it addresses an intervention that was codesigned with family members, the engagement of families is immediately relevant. In these cases, family members can productively advise on the research questions, processes and findings. However, if a study is addressing the experiences of PWLE, without a family component, or if PWLE can fully represent their experiences without the need for family perspectives, the relevance of engaging family members is not as immediately apparent. It is important to recognize that family members invest considerable personal time, effort, and emotional labour into the engagement process.^5^ It is therefore essential to focus engagement efforts on projects in which family voices are appropriate, needed, welcomed, and valued.

## 3 REPRESENTATIVENESS OF FAMILIES

Across engagement, it is important that the people engaged are representative of the target population. Definitions of families are diverse, including chosen families, family structures beyond the nuclear household, and families with diverse gender and sexual characteristics. It is important to consider the representativeness of families engaged across equity, diversity, and inclusion considerations, with trauma‐informed approaches.^6^ Many people with mental health or substance use challenges have supportive families of origin or families of choice who wish to be engaged in both their care and their lives. The families who come forward to be engaged often fit this profile and are enthusiastic contributors to our work. However, this profile is not representative of all people with mental health or substance use challenges. Indeed, family‐based violence, trauma and family conflict are directly associated with mental illness. Conflictual versus nonconflictual family dynamics are not a binary concept, but rather a complex reality among the population in general, including people with mental health or substance use challenges. Researchers working clinically may be very aware of complex family histories among their patients and may therefore inadvertently bring bias and stigma into engagement spaces. The family members who come forward to be engaged can feel stigmatized, blamed, and shamed, despite their wish to help; after first experiencing this as they help their loved ones navigate the healthcare system, they can re‐experience it in engagement processes. To avoid restigmatizing family members, researchers are called on to recognize them as supportive families who want to help, while acknowledging the stigma that comes with conflictual family dynamics. If supportive family dynamics are not representative of at least some of target population, family engagement might not be appropriate for a study.

## 4 FAMILY IS WELCOMED BY ALL STAKEHOLDERS

To experience research engagement as positive, family members need to be welcomed in the research space by all stakeholders. Researchers must be willing to establish equitable partnerships with them and a shared sense of purpose, using a family‐centred approach to engagement. Researchers have to be trained in strong engagement practices and be open to family feedback on their research, even if it does not align with their immediate goals and perspectives; they also have to be willing to negotiate a shared understanding and perspective, all within the context of sometimes rigid scholarly, institutional, funding, and approval contexts. In addition, if the study team is engaging PWLE, it is important that the PWLE want families to be engaged and consider their input helpful. This might not always be the case, for example, in contexts of family violence and conflict, or in key developmental stages such as adolescence. Despite the caregiving burden, PWLE may not always wish for family input. PWLE may sometimes feel that family voices overshadow their own, undermining their contributions to the research and their sense of autonomy in their own lives. To create a safe engagement space for family members, it is important that all team members, including research teams and PWLE, reflect on any biases they have that may be a barrier to authentic family engagement, that they truly want and need families around the table to address study issues relevant to them, that they welcome them there, and that they value their direct contributions to the relevant aspects of the study.

## 5 CONCLUSION

In a climate of increasing emphasis on engaging PWLE/F in research, some researchers may embark on family engagement processes because they believe family engagement is generally considered desirable. This can occur without due consideration of the circumstances and context of their specific research project and of the PWLE and family members they plan to engage. However, it is important to conceptualize this engagement in a thoughtful manner.^3^ When engagement is not thoughtful and reflective, there is a high risk of tokenization, leaving family members wondering why they are present and what their contributions might be. This can create unequal power dynamics and limited capacity for meaningful change, reflecting tokenistic engagement that advances neither the research nor the goal of authentic and antioppressive practice. Unauthentic engagement stands the risk of restigmatizing and retraumatizing families, creating environments that detract from authentic engagement and losing family members in the engagement process. By reflecting on the aim, purpose and goals of family engagement, as well as the engagement context, researchers can avoid engaging tokenistically and move towards the authentic, meaningful engagement of families, in the appropriate studies, enhancing research and creating positive experiences for all involved.

## AUTHOR CONTRIBUTIONS

Lisa D. Hawke conceptualized and drafted the paper. Connie Putterman further conceptualized the paper, edited the content, and approved the final version. Nathan Dawthorne, Shannon Pascoe and Shaylene Pind contributed to the content of the discussion, edited the manuscript, and approved the final version [Correction added on 14 August 2023, after first online publication: The preceding sentence has been corrected in this version.]
